# Nutrition Risk Assessment Using the Modified NUTRIC Score in Cirrhotic Patients with Acute Gastroesophageal Variceal Bleeding: Prevalence of High Nutrition Risk and its Independent Prognostic Value

**DOI:** 10.3390/nu11092152

**Published:** 2019-09-09

**Authors:** Ming-Hung Tsai, Hui-Chun Huang, Yun-Shing Peng, Yung-Chang Chen, Ya-Chung Tian, Chih-Wei Yang, Jau-Min Lien, Ji-Tseng Fang, Ming-Chih Hou, Chien-Heng Shen, Chung-Chi Huang, Cheng-Shyong Wu, Fa-Yauh Lee

**Affiliations:** 1 Division of Gastroenterology and Hepatology, Chang Gung Memorial Hospital, Chang Gung University, Taoyuan 333, Taiwan (M.-H.T.) (J.-M.L.); 2 Division of Gastroenterology and Hepatology, Department of Medicine, Taipei Veteran General Hospital, Faculty of Medicine, Yang-Ming University School of Medicine, Taipei 112, Taiwan; 3 Division of General Medicine, Department of Medicine, Taipei Veteran General Hospital, Faculty of Medicine Yang-Ming University School of Medicine, Taipei 112, Taiwan; 4 Division of Endocrinology and Metabolism, Chang Gung Memorial Hospital, Chia-Yi 613, Taiwan; 5 Division of Critical Care Nephrology, Kidney Institute, Chang Gung Memorial Hospital, Taipei 105, Taiwan (Y.-C.C.) (Y.-C.T.) (C.-W.Y.) (J.-T.F.); 6 Division of Gastroenterology, Chang Gung Memorial Hospital, Chia-Yi 613, Taiwan (C.-H.S.) (C.-S.W.); 7 Division of Critical Care Medicine, Chang Gung Memorial Hospital, Chang Gung University, Taoyuan 333, Taiwan

**Keywords:** nutrition risk, cirrhosis, variceal bleeding, ICU

## Abstract

Malnutrition is associated with adverse outcomes in patients with liver cirrhosis. Relevant data about nutrition risk in critically ill cirrhotic patients are lacking. The modified Nutrition Risk in Critically Ill (mNUTRIC) score is a novel nutrition risk assessment tool specific for intensive care unit (ICU) patients. This retrospective study was conducted to evaluate the prevalence and prognostic significance of nutrition risk in cirrhotic patients with acute gastroesophageal variceal bleeding (GEVB) using mNUTRIC scores computed on admission to the intensive care unit. The major outcome was 6-week mortality. One-hundred-and-thirty-one admissions in 120 patients were analyzed. Thirty-eight percent of cirrhotic patients with acute GEVB were categorized as being at high nutrition risk (a mNUTRIC score of ≥5). There was a significantly progressive increase in mortality associated with the mNUTRIC score (χ^2^ for trend, *p* < 0.001). By using the area under a receiver operating characteristic (ROC) curve, the mNUTRIC demonstrated good discriminative power to predict 6-week mortality (AUROC 0.859). In multivariate analysis, the mNUTRIC score was an independent factor associated with 6-week mortality. In conclusion, the mNUTRIC score can serve as a tool to assess nutrition risk in cirrhotic patients with acute GEVB.

## 1. Introduction

Critical illness is characterized by inflammation and neuroendocrine stress responses, which lead to catabolic response and deterioration of nutrition status [1]. Over the past years, accumulating lines of evidence have shown that varying degrees of inflammation contribute to the pathogenesis of malnutrition and hinder the effects of nutrition intervention in critical illness [1]. Indeed, a conceptual model has been proposed to link starvation, inflammation, malnutrition, and poor prognosis [2]. It has been shown that patients at higher risk of malnutrition benefit more from nutrition intervention than those who are at lower risk [2,3,4]. Although nutritional requirements and the optimal timing of intervention in intensive care unit (ICU) patients are still poorly understood, nutrition assessment has been considered as an integral part of critical care.

While many risk-stratifying scores exist to assess nutrition risk, few have been specifically designed and validated for ICU patients [5,6,7,8,9]. Traditional tools to evaluate nutrition status include a history of food intake and weight loss [7,8,9], which may be difficult to obtain or lack validity in ICU patients who are unconscious or on life support. Variations in body weight can be affected by fluid resuscitation that is often necessary to maintain hemodynamic stability in critical care, consequently making muscle wasting evaluation difficult. Furthermore, it is difficult to compare different studies because of various tools and criteria used to define malnutrition. In this regard, heterogeneous groups of ICU patients should be properly risk-stratified to optimize nutrition support. Recently, the Nutrition Risk in Critically Ill (NUTRIC) score, a novel risk assessment tool specific for ICU patients, was first proposed [2]. The NUTRIC score incorporates age, severity of disease reflected by the Acute Physiology and Chronic Health Evaluation II (APACHE II) and Sequential Organ Failure (SOFA) scores, co-morbidities, days from hospital to ICU admission, and inflammation assessed by the level of interleukin 6 (IL-6) [2]. This score was subsequently modified by deleting IL-6 levels to increase clinical utility because IL-6 is not routinely measured in clinical settings [4]. Both mNUTRIC and NUTRIC scores are prognostically significant and can identify ICU patients who may benefit from nutritional support [2,4]. Taken together, the modified NUTRIC score is easy to compute and may serve as a more useful tool because it contains variables which are readily available in the critical care setting. In fact, American Society for Parenteral and Enteral Nutrition (ASPEN) guidelines endorse the use of the NUTRIC score to assess nutrition risk in the ICU [10]. 

Recently, nutrition in patients with liver cirrhosis has drawn more attention and become an area of active investigation. Cirrhotic patients are characterized by various degrees of inflammation that may contribute to malnutrition [11,12]. It has been shown that malnutrition is associated with progression of liver insufficiency and higher rates of complications, including infection, ascites, and hepatic encephalopathy [13,14,15]. Importantly, malnutrition is an independent factor to predict poor prognosis in cirrhotic patients who are hospitalized or are on the liver transplant waiting list [16,17]. In critically ill cirrhotic patients, adequate nutrition support has been considered as a relevant target [12]. In terms of nutrition support, patients with liver cirrhosis and acute gastroesophageal variceal bleeding (GEVB) represent a unique subset. Oral or enteral feeding may be temporarily contraindicated because continuing to provide feeding in the early phase of acute GEVB may increase the likelihood of re-bleeding. Taken together, patients with liver cirrhosis and GEVB inevitably experience acute starvation in addition to pre-existing inflammation. Both are key factors that constitute the conceptual model of malnutrition in critical care. Finally, we need reliable scores to characterize those critically ill cirrhotic patients included in clinical trials that evaluate nutrition interventions. While nutrition risk assessment in this specific setting is an important issue, relevant data are lacking. We hypothesized that a higher nutrition risk is associated with poor outcomes in cirrhotic patients with acute GEVB. Therefore, we conducted this retrospective study to evaluate the prevalence and prognostic significance of nutrition risk in cirrhotic patients with acute GEVB using the mNUTRIC score. 

## 2. Materials and Methods

### 2.1. Patient Information, Data Collection, and Definitions

This study is a retrospective analysis of a prospective observational study of acute GEVB in patients with liver cirrhosis. This study was conducted with approval from the institutional review board of Chang Gung Memorial Hospital (IRB: 98-3658A3), Taiwan, and in accordance with the Declaration of Helsinki of the World Medical Association. Written informed consent was obtained from the patients or from their legally accepted representatives in those with hepatic encephalopathy. Between December 2011 and April 2013, cirrhotic patients presenting with acute GEVB admitted to ICU were considered for enrollment in the study. Liver cirrhosis was defined histologically or based on clinical, imaging, and laboratory findings. Acute GEVB was confirmed by emergency endoscopy according to Baveno consensus [18]. Patients with advanced hepatocellular carcinoma (HCC) (a single nodule of more than 5 cm or three nodules with one greater than 3 cm, or more than three nodules) were excluded. All patients were managed by the standard methods [18,19,20], including initial resuscitation, a restrictive strategy of blood transfusion as clinically indicated to maintain hemoglobin levels at approximately 7–8 g/dL [21], and antibiotic prophylaxis. All the patients were treated with a combination of vasoactive drugs from admission and early endoscopic therapy using band ligation for bleeding esophageal varices or tissue glue injection for bleeding gastric varices (within 12 h from admission). Main outcomes analyzed 6-week mortality. Five-day treatment failure was defined as death or by one of the following [18], whichever occurred first: (1) fresh hematemesis or nasogastric aspiration of more than 100 mL of fresh blood more than 2 h after the start of a specific drug treatment or therapeutic endoscopy; (2) development of hypovolemic shock; or (3) a 3 g drop in hemoglobin within any 24-h period if no transfusion was administered. Hypovolemic shock was defined by signs of peripheral hypoperfusion on physical examination, along with a systolic blood pressure below 100 mm Hg. Re-inclusion was only allowed if a separate bleeding episode occurred at least 42 days after the previous inclusion.

### 2.2. Laboratory Investigations

Hematological and biochemical studies, blood cultures, urine sediment, urine culture, and ascitic fluid neutrophil count and culture were routinely performed at inclusion. For those patients whose bleeding episodes happened when they had been hospitalized (*n* = 10), results of microbiological cultures were traced back to 5 days prior to inclusion.

### 2.3. Nutrition Risk Assessment and Disease Severity Scores

Nutrition risk was assessed using the mNUTRIC score [10]. Baseline clinical characteristics for computing mNUTRIC score included age, admission category (medical, elective, and non-elective surgery), number of comorbidities, APACHE-II score, SOFA-score, and duration of hospitalization prior to ICU admission. As previously described [2,4], patients with a score of 5 or more were considered as at high nutrition risk.

Meanwhile, the severity of liver disease was graded by the Child–Pugh system and the Model for End-stage Liver Disease (MELD) [22,23]. For these scoring systems and physiological evaluations, the most abnormal value for each organ system on the first day of ICU admission was recorded.

### 2.4. Statistical Analysis

Descriptive statistics are expressed as mean ± SD. All variables were tested for normal distribution using the Kolmogorov–Smirnov test. The Student’s *t*-test was used to compare the means of continuous variables and the normal distribution data. Otherwise, the Mann–Whitney *U* test was used. Categorical data were tested using the Chi-square (χ^2^) test. The time of survival were analyzed by the Kaplan–Meier method and compared between groups with the log-rank test. Meanwhile, risk factors were assessed using univariate analysis, and variables that were statistically significant in the univariate analysis were selected into the multivariate analysis by using a logistic regression to obtain independent risk factors. The associations between the results of C-reactive protein (CRP), Child–Pugh, and MELD scores and mNUTRIC were analyzed with linear regression using the Pearson method. Discrimination was tested using the area under a receiver operating characteristic (ROC) curve to assess the ability of mNUTRIC score to predict 6-week mortality. ROC analysis was also performed to calculate the cutoff values, sensitivity, specificity, overall correctness, and positive and negative predictive values. The best Youden index (sensitivity + specificity −1) was also used to determine the best cutoff point of mNUTRIC to predict 6-week mortality. All statistical tests were two-tailed, and the significance level was set at *p* = 0.05 or less. Data were analyzed using SPSS 13.0 for Windows (SPSS Inc., Chicago, IL, USA).

## 3. Results

**Figure 1:**
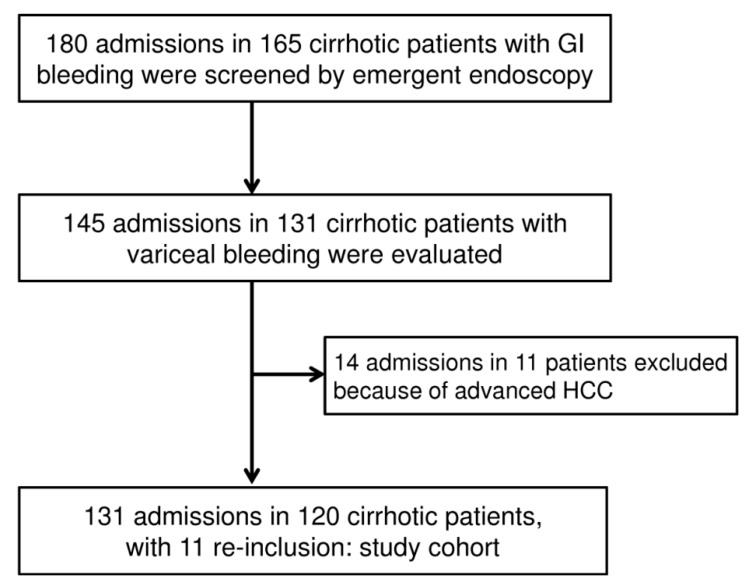
Diagram showing the flow of patients included in the study cohort. In 10 cases of study cohort, variceal bleeding episode occurred during the period of being hospitalized.

During the period of investigation, a consecutive series of 180 admissions in 165 cirrhotic patients with gastrointestinal (GI) bleeding took place in the ICU. The diagram in Figure 1 shows the flow of patients included in the study cohort.

### mNUTRIC Score and Outcomes

**Figure 2:**
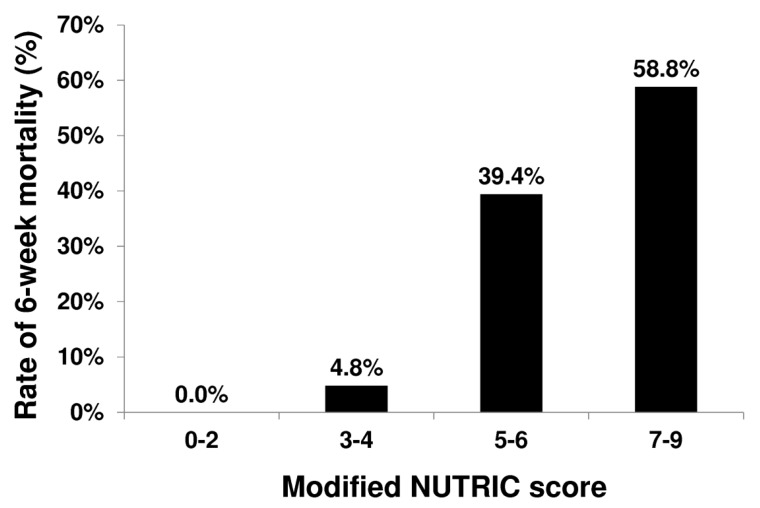
Relationship between 6-week mortality and mNUTRIC score (χ^2^ for trend, *p* < 0.001).

Table 1 shows the clinical characteristics and outcomes in patient subgroups stratified by 6-week outcome. There was a significantly progressive increase in mortality associated with the mNUTRIC (χ^2^ for trend, *p* <0.001, Figure 2). To identify the independent factors associated with 6-week mortality, a total of 25 variables in the baseline characteristics (Table 1) were analyzed for prognostic value. In univariate analysis, the variables with prognostic value are as shown in Table 2. In multivariate analysis, we excluded those variables that were indeed different operationalizations of the same concepts. We included mNUTRIC score, mean arterial pressure, bacterial infection at inclusion, and Child–Pugh score into the multivariate analysis (Model 1). mNUTRIC score, bacterial infection at inclusion and Child–Pugh score were independent factors predicting 6-week mortality (Table 2). We replaced Child–Pugh score with the MELD score, another major assessment tool for liver function, in Model 2. In this model, mNUTRIC score, bacterial infection at inclusion, and MELD score were independently associated with 6-week mortality.

The discriminating power of mNUTRIC score to predict 6-week mortality was tested using the area under a receiver operating characteristic (ROC) curve. The area under ROC curve for mNUTRIC score was 0.859 ± 0.035 (mean ± standard error; 95% confidence interval (95%CI): 0.790 to 0.928). The cut-off value for mNUTRIC to predict 6-week mortality was obtained by analyzing the ROC. The predictive values of the chosen cutoff points (4.5), which give the best Youden index (0.665), are as follows: sensitivity, 0.92; specificity, 0.745. The mNUTRIC scores were positively associated with CRP (R = 0.364, *p* <0.001), MELD (R = 0.479, *p* <0.001), and Child–Pugh scores (R = 0.390, *p* <0.001).

**Figure 3:**
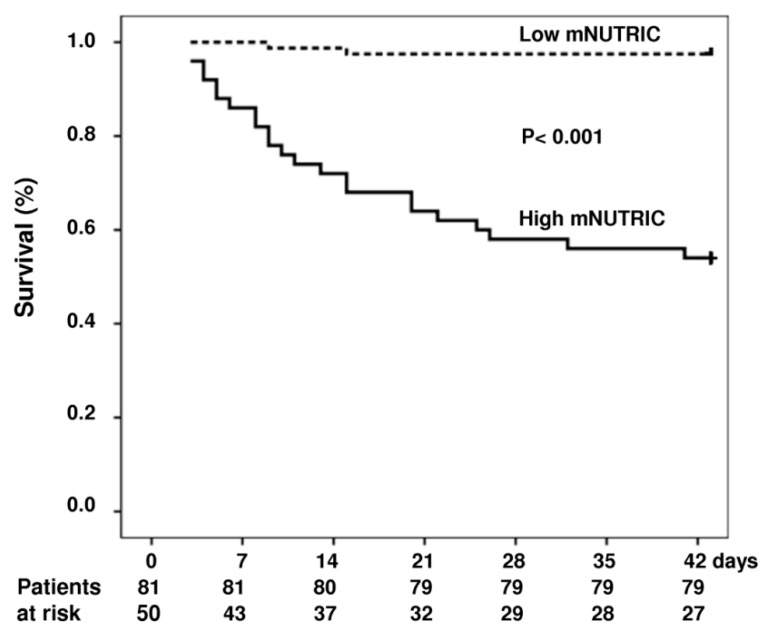
Cumulative survival in patients with and without adrenal insufficiency (dash curve, low mNUTRIC score group, *n* = 81; solid curve, high mNUTRIC group, *n* = 50). Probability of survival was significantly lower in patients with high mNUTRIC.

The ICU, 28-day and 6-week mortality rates were significantly higher in high nutrition risk group. Table 3 lists the demographic data, clinical characteristics for both low mNUTRIC and high mNUTRIC groups. Follow-up to 6 weeks or the time of death was complete for the entire groups. The cumulative rates of survival at 6 weeks were 97% and 54% for the low mNUTRIC group and high mNUTRIC group, respectively (*p* <0.001) (Figure 3).

Treatment failure rates for patients at high nutrition risk were significantly higher than those with low nutrition risk. Table 4 lists the demographic data and clinical characteristics for patients grouped according to treatment failure.

## 4. Discussion

The nutrition assessment in ICU patients presents a special challenge to intensivists. It may be especially difficult to find an optimal tool to evaluate nutrition status in critically ill cirrhotic patients because many of the traditional nutritional parameters, such as body weight and biochemical tests, may vary with the severity of liver disease independently of nutrition status. In this regard, the mNUTRIC score is a novel instrument specific for critically ill patients. Our study represents the first validation of the mNUTRIC score in patients with liver cirrhosis. Our major findings are as follows. Firstly, 38% of cirrhotic patients with acute GEVB were categorized as being at high nutrition risk (a mNUTRIC score of ≥5). Secondly, the group of high nutrition risk was characterized by higher levels of CRP, poor liver reserve, a longer ICU stay, and higher rates of mortality. Thirdly, the mNUTRIC score is an independent factor predicting 6-week mortality.

Recent studies have suggested that various degrees of acute or chronic inflammation are important factors contributing to the pathogenesis of malnutrition. Recognizing the role of inflammation, Jensen proposed an etiology-based approach to malnutrition syndrome [24], including three categories: (1) starvation-associated malnutrition, when there is chronic starvation without inflammation; (2) chronic disease-associated malnutrition, when inflammation is chronic and of mild to moderate degree; and (3) acute disease or injury-associated malnutrition, when inflammation is acute and of a severe degree. Across the category, the nutrition need increases while the responsiveness to nutrition support decreases progressively.

In terms of etiology-based approach, cirrhotic patients with acute GEVB represent a unique subgroup that deserves special attention. Cirrhotic patients with GEVB inevitably have acute starvation superimposing antecedent inflammation of various degrees. While enteral nutrition is recommended to ICU patients who have a functioning gastrointestinal tract [10], experts recommend withholding feeding for 2–3 days after acute bleeding [25,26], based on the assumption that early initiation of enteral feeding may increase splanchnic blood flow and consequently lead to variceal re-bleeding. Indeed, in those cirrhotic patients with bacterial infection who are at risk of treatment failure and rebleeding, prolonged fasting can be expected. Extended periods of starvation superimposed on an inflammation state are very likely to contribute to adverse outcomes. Taken together, cirrhotic patients with acute GEVB may readily progress from one malnutrition category to another. Therefore, early recognition of nutrition risk and frequent follow-up of assessment are relevant. In this regard, we demonstrated that high mNUTRIC scores were associated with high levels of CRP and higher rates of mortality, supporting the concept that an inflammation state contributes to malnutrition and a poor prognosis. While severe and overwhelming inflammation can be easily discerned, CRP may serve to recognize that of a lesser degree which is obscure and/or recurrent. In the context of critical care, CRP has an advantage over IL-6 because CRP is readily available in clinical settings. It is unknown whether sequential assessment of the mNUTRIC score plus CRP levels can assist in early recognition of emerging nutrition risk and timely nutrition intervention in cirrhotic patients with acute GEVB.

Considering the association between high nutrition risk and poor prognosis in acute GEVB, well-designed clinical trials are needed to clarify whether nutrition therapy can improve outcomes, especially in the most vulnerable subgroups, for example in patients categorized as Child–Pugh B and C.

Although optimal nutrition regimens and timing of interventions are still unknown, we hypothesize that mNUTRIC scores may help risk stratification and predict the effectiveness of nutrition intervention in clinical trials in this setting. While postprandial splanchnic hyperemia associated with initiation of feeding has been a concern in the acute stage of GEVB, timely nutrition therapy may ensure energy adequacy and protein intake without increasing portal pressure when vasoactive drugs are used concomitantly to counterbalance the potential adverse effects of feeding. These issues need to be addressed in the future.

There are limitations in our study. First, the present study is a retrospective analysis. Second, we did not assess the effects of nutritional adequacy on mortality in patients with regard to nutrition risk.

In conclusion, the high mNUTRIC scores are associated with high levels of CRP, impaired liver reserve, and poor outcomes in cirrhotic patients with acute GEVB. The mNUTRIC score is independently associated with 6-week mortality and can serve as a tool to evaluate nutrition risk in this clinical setting.

## Figures and Tables

**Table 1:** Patients’ demographic data and clinical characteristics grouped according to 6-week mortality.

	All (*n* = 131)	6-Week Mortality (*n* = 25)	6-Week Survival (*n* = 106)	*p*-value
Age	54.45 ± 14.20	53.88 ± 13.66	54.58 ± 14.39	NS (0.842)
Sex (male/female)	109/22	20/5	89/17	NS (0.634)
Etiology (alcohol/virus/mixed type/others)	51/67/12/1	13/10/2/0	38/57/10/1	NS (0.550)
mNUTRIC score	3.88 ± 2.22	6.27 ± 1.39	3.32 ± 2.01	<0.001
High mNUTRIC score	50/131 (38.2)	23/25 (92.0)	27/106 (25.5)	<0.001
BMI	24.12 ± 4.24	24.41 ± 5.01	24.05 ± 4.06	NS (0.729)
Child–Pugh score	9.65 ± 2.34	12.48 ± 1.74	8.98 ± 1.93	<0.001
Child A/B/C	12/51/68	0/1/24	12/50/44	<0.001
SOFA score	7.57 ± 3.55	9.84 ± 3.83	6.65 ± 2.98	<0.001
APACHE II	20.57 ± 8.34	29.68 ± 8.76	18.42 ± 6.64	<0.001
MELD	18.92 ± 10.23	31.88 ± 11.48	15.86 ± 7.07	<0.001
CRP (mg/L)	37.30 ± 38.00	60.41 ± 52.82	32.00 ± 31.84	0.003
Albumin (g/dL)	2.59 ± 0.55	2.42 ± 0.61	2.63 ± 0.52	NS (0.080)
Bilirubin (mg/dL)	5.28 ± 7.62	13.49 ± 13.08	3.35 ± 3.61	<0.001
INR	1.81 ± 1.18	2.90 ± 2.15	1.55 ± 0.38	<0.001
Serum creatinine (mg/dL)	1.68 ± 1.49	2.82 ± 1.83	1.41 ± 1.27	<0.001
Hemoglobulin (g/dL)	8.43 ± 1.55	8.58 ± 1.96	8.41 ± 1.48	NS (0.673)
Platelet (×10^9^/L)	65.02 ± 35.49	56.08 ± 32.66	67.15 ± 35.95	NS (0.162)
Leukocytes (×10^9^/L)	9.43 ± 6.08	12.25 ± 9.01	8.75 ± 4.96	0.009
Blood transfusion (unit)	4.56 ± 4.01	8.75 ± 5.04	3.65 ± 3.13	<0.001
Mean arterial pressure (mmHg)	73.91 ± 12.87	64.22 ± 11.12	76.31 ± 12.17	0.003
Shock (%)	59/131 (45.0)	22/25 (88.0)	37/106 (34.9)	<0.001
Ascites (%)	83/131 (63.4)	24/25 (96.0)	59/106 (55.7)	0.001
Hepatic encephalopathy (%)	73/131 (55.7)	20/25 (80.0)	53/106 (50.0)	0.007
Hepatocellular carcinoma (%)	33/131 (25.2)	9/25 (36.0)	24/106 (22.6)	NS (0.166)
Bacterial infection at inclusion (%)	42/131 (32.1)	19/25 (76.0)	23/106 (21.7)	<0.001
Treatment failure (%)	38/131 (29.0)	20/25 (80.0%)	18/106 (17.0%)	<0.001
ICU stay (days)	8.05 ± 8.54	12.76 ± 11.04	6.91 ± 7.47	0.002

Abbreviations: mNUTRIC, Modified Nutrition Risk in the Critically Ill; BMI, body mass index; SOFA, Sequential Organ Failure Assessment; APACHE II, Acute Physiology and Chronic Health Evaluation II; MELD, Model for End-Stage Liver Disease; CRP, C-reactive protein; INR, international normalized ratio; ICU, intensive care unit; NS, non-significant.

**Table 2:** Multivariate analysis to predict 6-week outcomes.

Parameter	Beta Coefficient	Standard Error	Odds Ratios (95%CI)	*p*
**Univariate logistic regression**				
mNUTRIC score	0.849	0.175	2.338 (1.658–3.296)	<0.001
High mNUTRIC score	3.516	0.770	33.648 (7.437–152.243)	<0.001
Child–Pugh score	1.007	0.200	2.738 (1.850–4.053)	<0.001
SOFA score	0.586	0.115	1.796 (1.434–2.250)	<0.001
APACHE II	0.176	0.036	1.192 (1.112–1.278)	<0.001
MELD	0.175	0.033	1.191 (1.116–1.272)	<0.001
CRP (mg/L)	0.017	0.006	1.017 (1.005–1.030)	0.007
Bilirubin (mg/dL)	0.180	0.044	1.197 (1.098–1.305)	<0.001
INR	3.044	0.625	20.980 (6.157–71.481)	<0.001
Serum creatinine (mg/dL)	0.528	0.138	1.695 (1.293–2.223)	<0.001
Leukocytes (×10^9^/L)	0.082	0.035	1.085 (1.014–1.162)	0.0185
Blood transfusion (unit)	0.291	0.079	1.337 (1.146–1.561)	<0.001
Mean arterial pressure (mmHg)	−0.096	0.025	0.908 (0.865–0.953)	<0.001
Shock (%)	2.616	0.648	13.676 (3.838–48.730)	<0.001
Ascites (%)	2.951	1.039	19.119 (2.494–146.557)	0.005
Hepatic encephalopathy (%)	1.386	0.536	4.000 (1.398–11.446)	0.010
Bacterial infection at inclusion (%)	2.436	0.524	11.428 (4.090–31.929)	<0.001
Treatment failure (%)	2.973	0.563	19.556 (6.488–58.947)	0.001
ICU stay (days)	0.064	0.024	1.066 (1.017–1.117)	0.007
**Multivariate logistic regression (Model 1)**				
mNUTRIC score	0.770	0.238	2.160 (1.355–3.445)	0.001
Bacterial infection at inclusion	1.980	0.819	7.240 (1.453–36.071)	0.016
Child–Pugh score	0.954	0.274	2.596 (1.518–4.439)	<0.001
**Multivariate logistic regression (Model 2)**				
mNUTRIC score	0.875	0.292	2.399 (1.353–4.256)	0.003
Bacterial infection at inclusion	3.155	0.948	23.443 (3.659–150.178)	0.001
MELD score	0.184	0.052	1.202 (1.086–1.330)	<0.001

Abbreviations: mNUTRIC, Modified Nutrition Risk in the Critically Ill; BMI, SOFA, Sequential Organ Failure Assessment; APACHE II, acute physiology and chronic health evaluation II; MELD, Model for End-Stage Liver Disease; CRP, C-reactive protein; INR, international normalized ratio; ICU, intensive care unit; CI, confidence interval.

**Table 3:** Patients’ demographic data and clinical characteristics grouped according to modified NUTRIC score.

	All (*n* = 131)	High mNUTRIC (*n* = 50)	Low mNUTRIC (*n* = 81)	*p*-value
Age	54.45 ± 14.20	60.62 ± 13.51	50.64 ± 13.33	<0.001
Sex (male/female)	109/22	38/12	71/10	NS (0.083)
Etiology (alcohol/virus/mixed type/others)	51/67/12/1	17/30/3/0	34/37/9/1	NS (0.359)
BMI	24.12 ± 4.24	24.51 ± 4.21	23.87 ± 4.27	NS (0.448)
Child–Pugh score	9.65 ± 2.34	11.00 ± 2.28	8.81 ± 1.97	<0.001
Child A/B/C	12/51/68	1/12/37	11/39/31	<0.001
SOFA score	7.57 ± 3.55	10.40 ± 3.42	5.83 ± 2.29	<0.001
APACHE II	20.57 ± 8.34	27.90 ± 7.47	16.05 ± 4.95	<0.001
MELD	18.92 ± 10.23	26.22 ± 11.04	14.41 ± 6.44	<0.001
CRP (mg/L)	37.30 ± 38.00	53.97 ± 43.46	26.09 ± 29.21	<0.001
Albumin (g/dL)	2.59 ± 0.55	2.49 ± 0.53	2.65 ± 0.55	NS (0.091)
Bilirubin (mg/dl)	5.28 ± 7.62	7.69 ± 9.88	3.80 ± 5.37	0.004
INR	1.81 ± 1.18	2.24 ± 1.67	1.54 ± 0.38	<0.001
Serum creatinine (mg/dL)	1.68 ± 1.49	2.68 ± 1.84	1.06 ± 0.72	<0.001
Hemoglobulin (g/dL)	8.43 ± 1.55	8.45 ± 1.51	8.42 ± 1.59	NS (0.924)
Platelet (x10^9^/L)	65.02 ± 35.49	63.53 ± 33.08	65.92 ± 37.04	NS (0.711)
Leukocytes (x10^9^/L)	9.43 ± 6.08	11.57 ± 7.76	8.12 ± 4.32	0.002
Blood transfusion (unit)	4.56 ± 4.01	5.17 ± 4.71	4.15 ± 3.44	NS (0.243)
Mean arterial pressure (mmHg)	73.91 ± 12.87	68.24 ± 11.82	77.41 ± 12.30	<0.001
Shock (%)	59/131 (45.0)	28/50 (56.0)	31/81 (38.3)	0.048
Ascites (%)	83/131 (63.4)	39/50 (78.0)	44/81 (54.3)	0.006
Hepatic encephalopathy (%)	73/131 (55.7)	33/50 (66.0)	40/81 (49.4)	NS (0.063)
Hepatocellular carcinoma (%)	33/131 (25.2)	21/60 (42.0)	12/81 (14.8)	<0.001
Bacterial infection at inclusion (%)	42/131 (32.1)	23/50 (46.0)	19/81 (23.5)	0.007
***Outcome***				
Treatment failure (%)	38/131 (29.0)	24/50 (48.0)	14/62 (17.3)	<0.001
ICU stay (days)	8.05 ± 8.54	11.20 ± 10.56	6.05 ± 6.26	0.001
ICU mortality (%)	26/131 19.8)	23/50 (46.0)	3/81 (3.7)	<0.001
28-day mortality (%)	23/131 (17.6)	21/50 (42.0)	2/81 (2.5)	<0.001
6-week mortality (%)	25/131 (19.1)	23/50 (46.0)	2/81 (2.5)	<0.001

Abbreviations: mNUTRIC, modified nutrition risk in the critically ill; BMI, body mass index; SOFA, Sequential Organ Failure Assessment; APACHE II, Acute Physiology and Chronic Health Evaluation II MELD, Model for End-Stage Liver Disease; CRP, C-reactive protein; INR, international normalized ratio; ICU, intensive care unit.

**Table 4:** Patients’ demographic data and clinical characteristics at admission to ICU grouped according to treatment failure.

	All (*n* = 131)	Treatment Failure (*n* = 38)	Treatment Success (*n* = 93)	*p*-value
Age	54.45 ± 14.20	52.18 ± 11.54	55.38 ± 15.12	NS (0.245)
Sex (male/female)	109/22	34/4	75/18	NS (0.220)
Etiology (Alcohol/virus/mixed type/others)	51/67/12/1	20/14/3/1	31/53/9/0	NS (0.090)
mNUTRIC score	3.88 ± 2.22	4.82 ± 2.48	3.49 ± 2.00	0.002
High mNUTRIC score	50/131 (38.2)	24/38 (63.2)	26/93 (28.0)	<0.001
BMI	24.12 ± 4.24	25.01 ± 4.29	23.72 ± 4.18	NS (0.147)
Child–Pugh score	9.65 ± 2.34	10.87 ± 2.27	9.15 ± 2.19	<0.001
Child A/B/C	12/51/68	0/12/26	12/39/42	<0.001
SOFA score	7.57 ± 3.55	10.40 ± 3.42	5.83 ± 2.29	<0.001
APACHE II	20.57 ± 8.34	24.39 ± 9.88	19.01 ± 7.10	0.001
MELD	18.92 ± 10.23	24.58 ± 13.03	16.60 ± 7.80	<0.001
CRP (mg/L)	37.30 ± 38.00	49.57 ± 50.26	31.94 ± 30.08	0.030
Albumin (g/dL)	2.59 ± 0.55	2.43 ± 0.58	2.65 ± 0.52	0.035
Bilirubin (mg/dL)	5.28 ± 7.62	7.71 ± 10.62	4.29 ± 5.78	0.019
INR	1.81 ± 1.18	2.38 ± 1.87	1.57 ± 0.41	<0.001
Serum creatinine (mg/dL)	1.68 ± 1.49	2.25 ± 1.87	1.44 ± 1.25	<0.005
Hemoglobulin (g/dL)	8.43 ± 1.55	8.24 ± 1.73	8.50 ± 1.49	NS (0.423)
Platelet (x10^9^/L)	65.02 ± 35.49	57.09 ± 32.71	68.30 ± 36.24	NS (0.101)
Leukocytes (x10^9^/L)	9.43 ± 6.08	9.72 ± 5.87	9.31 ± 6.19	NS (0.728)
Blood transfusion (unit)	4.56 ± 4.01	7.84 ± 4.76	3.28 ± 2.81	<0.001
Mean arterial pressure (mmHg)	73.91 ± 12.87	68.56 ± 12.87	76.06 ± 12.30	0.003
Shock (%)	59/131 (45.0)	31/38 (81.6)	28/93 (30.1)	<0.001
Ascites (%)	83/131 (63.4)	32/38 (84.2.0)	51/93 (54.8)	0.002
Hepatic encephalopathy (%)	73/131 (55.7)	24/38 (63.2)	49/93 (52.7)	NS (0.274)
Hepatocellular carcinoma (%)	33/131 (25.2)	13/38 (34.2)	20/93 (21.5)	NS (0.128)
Bacterial infection at inclusion (%)	42/131 (32.1)	21/38 (55.3)	21/93 (22.6)	<0.001
***Outcome***				
ICU stay (days)	8.05 ± 8.54	10.11 ± 10.21	7.22 ± 7.67	NS (0.082)
ICU mortality (%)	26/131 (19.8)	19/38 (50.0)	7/93 (7.5)	<0.001
28-day mortality (%)	23/131 (17.6)	18/38 (47.4)	5/93 (5.4)	<0.001
6-week mortality (%)	25/131 (19.1)	20/38 (52.6)	5/93 (5.4)	<0.001

Abbreviations: mNUTRIC, Modified Nutrition Risk in the Critically Ill; BMI, body mass index; SOFA, Sequential Organ Failure Assessment; APACHE II, acute physiology and chronic health evaluation II; MELD, Model for End-Stage Liver Disease; CRP, C-reactive protein; INR, international normalized ratio; ICU, intensive care unit.
